# Demographical and Clinical Characteristics of Women with Provoked Vestibulodynia in Israel

**DOI:** 10.1177/26884844251387013

**Published:** 2025-10-09

**Authors:** Lea Tene, Adi Y. Weintraub, Leonid Kalichman

**Affiliations:** ^1^ Department of Physical Therapy, Recanati School for Community Health Professions, Faculty of Health Sciences, Ben-Gurion University of the Negev, Beer Sheva, Israel.; ^2^ Department of Obstetrics and Gynecology and Urogynecology Clinic, Soroka University Medical Center and Faculty of Health Sciences, Ben-Gurion University of the Negev, Beer Sheva, Israel.

**Keywords:** dyspareunia, vulvodynia, provoked vestibulodynia, pelvic floor, Israel

## Abstract

**Background:**

Provoked vestibulodynia (PVD) is a chronic pain condition affecting the vulvar vestibule, often triggered by minimal touch or pressure. It is underdiagnosed and poorly understood, leading to significant impacts on women’s quality of life.

**Objective:**

This study aims to identify the demographic and clinical characteristics of PVD among women in Israel.

**Materials and Methods:**

A cross-sectional study was conducted using an online questionnaire completed by a self-selected sample of adult women. Weighted estimates of vulvodynia prevalence and characteristics were determined.

**Results:**

Out of 250 participants, 196 women (78%) met the diagnostic criteria for PVD. High rates of comorbid conditions were observed, including depression (33%), anxiety (43.3%), and other comorbidities (60%). The mean time from first consulting a physician to diagnosis was 2.5 years, with symptoms persisting for an average of 8 years. Physical therapy emerged as the most effective treatment modality.

**Conclusions:**

PVD is a challenging condition to diagnose and treat, often leading to significant delays in diagnosis and prolonged suffering. The high prevalence of comorbid conditions underscores the need for comprehensive treatment approaches. Improved diagnostic protocols and more effective treatment options are essential to enhance the quality of life for women with PVD.

## Introduction

Provoked vestibulodynia (PVD) is defined as idiopathic pain in the vulva lasting for 3 or more months. The pain is confined to the vulvar vestibule and is often described as burning, stinging, stabbing, or tearing. It may be distributed throughout the entire vestibule or localized to its lower portion. Vestibular pain may be caused by minimal touch (*e.g.,* sitting or tight-fitting clothing). In the absence of touch or pressure, women are often symptom-free. Typically, pain is induced by sexual intercourse, tampon insertion, or gynecological examination. The condition is defined by it’s onset (primary or secondary); both can be provoked or unprovoked, and the pain can last for hours or days after intercourse. In severe cases, it can preclude sexual intercourse.^1,2^

Vulvodynia, a broader term for chronic vulvar pain, includes conditions like PVD, which is specifically characterized by pain in the vulvar vestibule. Research to date has yet to determine what causes PVD; its etiology is believed to be multifactorial. The Consensus Terminology and Classification of Persistent Vulvar Pain and Vulvodynia Report^2^ listed various factors associated with vulvodynia, including comorbidities and other pain syndromes (*e.g.,* painful bladder syndrome, fibromyalgia, irritable bowel syndrome, temporomandibular disorder), genetic and hormonal factors (*e.g.,* pharmacologically induced), inflammation, musculoskeletal disorders (*e.g.,* pelvic muscle overactivity, myofascial, biomechanical), neurological mechanisms, either central (spine, brain) or peripheral (neuroproliferation), psychosocial factors (*e.g.,* mood, interpersonal, coping, role, sexual function), and structural defects (*e.g.,* perineal descent).^2^

Research on the incidence and prevalence of vulvodynia is nascent. Spain, for example, has no epidemiological data on vulvodynia, and it remains underdiagnosed in the United States. The incidence of vulvodynia has been reported to range from 11% to 28%, with underreporting a known limitation to incidence rates.^3,4^ The cost of treating vulvodynia in the United States ranges between 31 and 72 billion dollars annually.^3^ Vulvodynia is the most common cause of sexual pain in premenopausal women,^5^ and its prevalence is consistent for sexually active women. The age range of women with symptoms of vulvodynia varies from 16 to 80 years, with the most common ages being between 20 and 60 years,^6,7^ with most pain being experienced^6^ and reported by young women.^4^ There is a second peak of vulvar pain prevalence around menopause, in part due to vulvovaginal atrophy and also new-onset or recurrent vulvodynia.^8^ It has been reported that 8% of menopausal women have localized pain in the vulvar vestibule (PVD), impacting sexual intercourse.^9^

Due to the lack of knowledge of the demographic and clinical characteristics of PVD, the aims of the present study were:

▪ To identify clinical and demographic characteristics of women with dyspareunia due to PVD in Israel.
▪ To evaluate associations between vestibulodynia and comorbidities such as functional impairment, sexual function disorders, and anxiety.
▪ To evaluate associations between sexual function (assessed by PISQ—Pelvic Organ Prolapse/Urinary Incontinence Sexual Questionnaire) and vestibulodynia.
▪ To evaluate utilization and satisfaction with physical therapy and alternative treatments.

## Methods

### Design

**FIG. 1.**
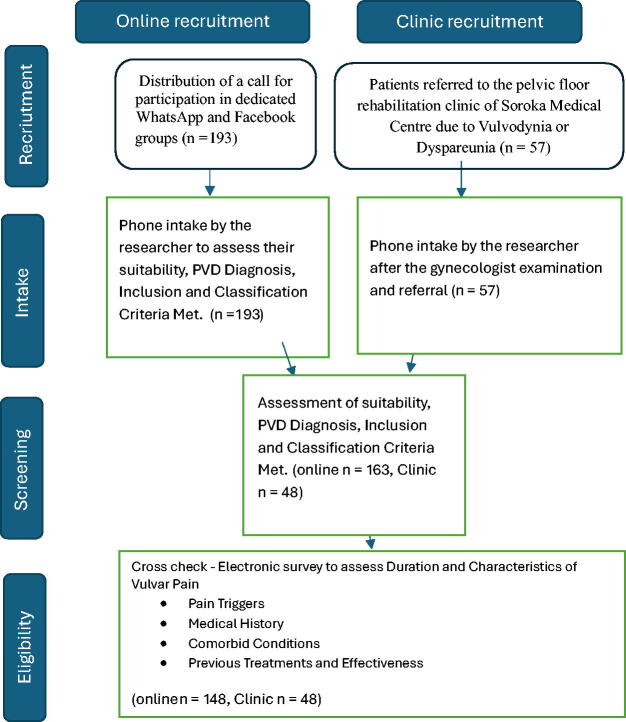
Study flowchart.

Cross-sectional quantitative observational study (Fig. 1).

### Sample

Women with PVD in Israel.

Inclusion criteria:

• Hebrew-speaking.
• Aged 18 years or older.
• Diagnosed with dyspareunia or any other pelvic floor sexual pain, specifically PVD.
• Possession of a smartphone.

Exclusion criteria:

• Current participation in a clinical trial.
• History of major psychiatric or neurological illness.
• History of significant trauma or surgery involving the genitourinary system.
• Presence of an active rheumatological disease.

### Sample size estimation

We used hierarchical regression to predict PVD pain. With up to 10 independent variables, a sample of more than 115 participants is required to identify medium to large effects (where α = 0.05 and *d* = 0.80).^10^

### Ethical considerations

Completion of the online questionnaire was voluntary. Prospective patients received a detailed description of the research and were asked to provide informed consent. All identifying details were number-coded and stored in password-protected computer files. This study received ethical approval from the Helsinki Committee of Soroka University Medical Center (MOH_2021-11-9_010383).

### Recruitment process

The recruitment for the study was conducted through two primary methods:

#### Online recruitment

A call for participation was distributed in dedicated Women’s WhatsApp groups as well as in Facebook groups for women with vulvodynia. Initially, 193 women responded to the announcement. The researcher contacted them and collected details to determine if they met the inclusion criteria, matched the classification of PVD, and had a diagnosis of PVD from a gynecologist. After this screening, 30 women were excluded because they did not meet one or more of the criteria or did not consent to participate in the study. This left 163 women. These women were summoned to a meeting where they underwent another examination by a gynecologist and were asked to consent. After this filtering, 15 more women dropped out, leaving 148 women. The announcement was circulated for 5 months and was reposted once a month.

#### Clinic recruitment

Fifty-seven women were recruited through the women’s clinic at Soroka Medical Center. These women visited the clinic due to pain during intercourse and were diagnosed with PVD by a gynecologist, meeting the inclusion and classification criteria. After a conversation with the researcher and an explanation of the study, nine women chose not to participate, leaving a total of 48 women.

#### Survey and diagnosis confirmation

An electronic survey hosted on Google Docs was sent to the eligible women. The survey included detailed questions to confirm the diagnosis of PVD, such as duration and characteristics of vulvar pain; pain triggers (*e.g.,* sexual intercourse, tampon insertion, gynecological examination); medical history, including any previous diagnoses of vulvodynia or dyspareunia; comorbid conditions (*e.g.,* irritable bowel syndrome [IBS], fibromyalgia); and previous treatments (*e.g.,* physical therapy) and their effectiveness.

The diagnosis of PVD was confirmed by reviewing the medical records of the women recruited from the hospital. For the 148 women recruited through social media, the diagnosis was based on their survey responses, which were cross-checked with any available medical documentation they provided.

#### Total recruitment

A total of 250 women were initially recruited through both methods. After the filtering process, 196 women remained in the study: 148 through online recruitment and 48 through the women’s clinic.

### Questionnaires

• Vulvar Pain Functional Questionnaire (VQ) was created for physical therapists to identify situations that exacerbate pelvic pain as well as to measure functional impairment. This tool aids the practitioner in deciding on the best treatment strategy. It has sufficient internal consistency (α = 0.75), and test–retest reliability over 4–6 weeks was high (*i.e.*, 87% agreement).^11^
• The health-related quality of life was evaluated by the SF-12 questionnaire,^12^ which has been widely used to assess the effectiveness of treatment for mental disorders in general practice, including the impact of illnesses on a broad range of functional domains. The SF-12 examines both physical well-being (*i.e.*, physical component summary [PCS]) and mental well-being (*i.e.*, mental component summary [MCS]). Scores are standardized, so a score lower than 50 indicates worse physical or mental health than the mean. By corollary, a score above 50 indicates better physical or mental health than average. Responses to a Hebrew version of SF-12 are valid and reliable; that is, high internal consistency for the PCS, 0.84 < α < 0.92; low to high internal consistency for the MCS, 0.63 < α < 0.83.^13^
• The Pelvic Organ Prolapse/Urinary Incontinence Sexual Questionnaire–12 (PISQ-12) is a condition-speciﬁc, self-administered scale measuring sexual function in patients with incontinence and/or uterovaginal prolapse. Responses to the Hebrew version of the PISQ-12 range from low to high internal consistency (0.65 < α < 0.96) and high test–retest reliability in a 2-week period (ρ = 0.81–0.98; *p* < 0.001).^14^
• Symptoms of anxiety were measured by the STAI (State-Trait Anxiety Inventory). This tool was developed by Spielberger^15^ and contained 20 questions.

### Statistical analysis

All statistical computations were performed using SPSS version 29.0 (IBM, Chicago, IL, USA). The significance level was set *a priori* at α < 0.05.

Descriptive statistics were used to characterize the sample. Quantitative and qualitative variables were presented as mean ± standard deviation (X ± SD) and percent (%), respectively.The univariate analyses were performed to assess the association between predictors and outcomes. Predictors associated with outcomes at level 0.1 were included in the final multivariate hierarchical linear regression analysis.

## Results

A total of 196 women were included in our study. Descriptive statistics are presented in Table 1. The mean age was 29.54 (20–44) years; the mean body mass index (BMI) was 23.55 (16.18–40.40) kg/m^2^, and 12.70% (25 women) were smokers. The mean time participating in physical activity was 1.66 hours per week (SD = 1.58). More than two-thirds had an academic degree (68.8%, 135 women).

**Table 1.** Descriptive Statistics

Variables	*N*	Mean *±* SD
Age (years)	196	29.54 ± 5.78
BMI (kg/m^2^)	196	23.55 ± 4.80
Time to diagnosis (years)	196	2.42 ± 1.90
Sport (times per week)	196	1.66 ± 1.58
Duration of PVD symptoms at the time of the survey (years)	196	8.09 ± 5.54
No. of physical therapy sessions	151	11.96 ± 14.70

	***N* (%)**	
Smoking	25 (12.70%)	
SSRI/SNRI	43 (22%)	
Contraception pills	22 (11%)	
Endometriosis	25 (12.70%)	
Fibromyalgia	19 (9%)	
Migraine	32 (16.30%)	
IBS	24 (12%)	
Depression	65 (33%)	
Anxiety	85 (43%)	
Sexual assault	39 (19.80%)	
Domestic violence	17 (8.60%)	

BMI, body mass index; IBS, irritable bowel syndrome; PVD, provoked vestibulodynia; SSRI, Selective Serotonin Reuptake Inhibitor; SNRI, Serotonin–Norepinephrine Reuptake Inhibitor.

Sixty-five women (33%) reported feeling depressed, and 85 (43%) reported feeling anxious, yet only 43 (22%) of these women were prescribed antidepressant or anxiolytic medication. Thirty-two women (16.30%) reported migraine, 24 (12.24%) with IBS, 19 (9.70%) had a diagnosis of fibromyalgia, and 25 (12.70%) had a diagnosis of endometriosis. The mean age when women in our study had their first sexual experience was 20 (14–35) years; 39 (19.8%) women had a previous sexual assault, and 17 (8.67%) reported domestic violence.

Most women reported primary vestibulodynia (59.2%). The mean time to diagnosis after onset of symptoms was 2.42 years (SD = 1.90, 0–6 years), and for most, conventional treatments did not provide major improvement: The vulvodynia symptoms persisted for many years (mean = 8.09 years, SD = 5.54).

Table 2 reports correlation coefficients between questionnaires used in this study. VQ showed a significant negative correlation with SF-12 (*r* = −0.19, *p* < 0.01), PISQ (*r* = −0.23, *p* < 0.01), and a positive correlation (*r* = 0.21, *p* < 0.01) with STAI. It means that women with more severe symptoms of PVD have lower health-related quality of life, poorer sexual function, and greater anxiety.

**Table 2.** Correlations Between Study Variables (*n* = 196)

Questionnaire	SF-12	STAI	PISQ
VQ	*r* = −0.19**	*r* = 0.21**	*r* = −0.23**
SF-12		*r* = −0.26**	*r* = −0.16*
STAI	—		*r* = 0.162*

*p < 0.05; **p < 0.01.

PISQ, Pelvic Organ Prolapse/Urinary Incontinence Sexual Questionnaire; STAI, State-Trait Anxiety Inventory; VQ, Vulvar Pain Functional Questionnaire.

Table 3 presents the satisfaction levels of various treatments for PVD as reported by the participants, using a Likert scale ranging from 1 to 3. The treatments evaluated include physiotherapy, topical creams, psychological therapy, laser treatment, hymenectomy, vestibulectomy, sexual consultation, and other alternative treatments such as acupuncture and tantra. One hundred and nineteen women were treated with some topical cream: only 10 (8.4%) reported any symptom improvement; 27 women were treated with laser, but only 8 reported any improvement; 16 women underwent a hymenectomy, and 7 a vestibulectomy operation, but only 5 and 1, respectively, describe any improvement. Forty-eight women consulted a sexual therapist, 20 (41.6%) reported improvement. Physical therapy seemed to be the most used and most effective treatment: 153 (78%) of the women were treated with a mean of 12 sessions of physical therapy, and yet, 30 (19%) women said it did not help them at all, 41% reported some improvement, and 39% reported a major improvement after these physical therapy sessions.

**Table 3.** Satisfaction Scale (1–3; 3 = Highest) for Different Treatment Modalities

Interventions	*N*	Mean *±* SD	Median
Physiotherapy	153	2.20 ± 0.74	2
Cream	119	1.10 ± 0.38	1
Psychology	74	1.60 ± 0.77	1
Laser	27	1.30 ± 0.46	1
Hymenectomy	16	1.50 ± 0.81	1
Vestibulectomy	7	1.14 ± 0.37	1
Sexual consultation	48	1.56 ± 0.74	1
Other (Acupuncture, Tantra)	82	2.73 ± 1.38	2

These results indicate that physical therapy and alternative treatments such as acupuncture and tantra received relatively higher satisfaction scores compared with other interventions.

### Hierarchical regression analysis

For this study, we performed a four-step hierarchical linear regression analysis predicting the severity of vestibulodynia. This ensured that additional variance explained in subsequent steps is unique to new variables and not shared with prior variables.

For the first step, demographic characteristics (current age, BMI, and the age at first sexual experience) were entered as predictors accounting for 7% of the observed variance in VQ scores (*R*^2^ = .07, *p* < 0.01). Yet of these demographic variables, only age at which one started to have sex, was statistically significant (β = −0.23, *p* < 0.001).

For the second step, we included behavioral factors (*i.e.,* smoker/nonsmoker, number of hours per week of physical activity, and number of sleeping hours per night), which accounted for no additional observed variance (Δ*R*^2^ = 0.003, *p* = 0.872); none of these variables were statistically significant.

For the third step, we included comorbid physical conditions and a history of abuse (endometriosis, IBS, migraine, fibromyalgia, anxiety, depression, history of sexual assault, and history of domestic violence). This step accounted for a further 9% of the variance in VQ scores (Δ*R*^2^ = 0.093, *p* = 0.012); endometriosis (β = 0.17, *p* = 0.038), fibromyalgia (β = 0.17, *p* = 0.040), and history of domestic violence (β = −0.15, *p* = 0.049) were each statistically significant.

For the fourth step, we included mental health variables (*i.e.,* SF-12, STAI, and PISQ-12), accounting for a further 6% variance in VQ scores. Only STAI scores were statistically significant (β = 0.18, *p* = 0.020).

This model accounted for less than a quarter of the observed variance in the severity of vestibulodynia (*R*^2^ = 0.229, *p* < 0.001). Although significant, the majority of variance in VQ scores remains unexplained by this model (Table 4). The unexplained variance in VQ scores may be due to individual differences in pain perception, psychological factors, or other unmeasured variables.

**Table 4.** Hierarchical Linear Regression Analysis of Predictors of Provoked Vestibulodynia

	B	β	F	Δ*R*^2^
Sociodemographic characteristics				0.07**
Age	−0.06	−0.07	−0.97	
Body mass index	0.04	0.04	0.58	
Age at first sexual experience	−0.31	−0.22	−3.11**	
Lifestyle variables				0.00
Non/smoker	−0.53	−0.03	−0.53	
# hours physical activity/week	0.01	0.00	0.05	
# hours sleep/night	−0.10	−0.02	−0.34	
Comorbid conditions				0.09*
Endometriosis	2.32	0.16	2.08*	
Irritable bowel syndrome	−0.17	−0.01	−0.18	
Migraine	−1.15	−0.08	−1.13	
Fibromyalgia	2.75	0.17	2.07*	
Anxiety	−0.18	−0.20	−0.23	
Depression	−0.14	−0.01	−0.18	
History of sexual assault	0.17	0.01	0.18	
History of domestic violence	−2.50	−0.15	−1.98*	
Mental health				0.06**
SF-12	−0.02	−0.12	−1.63	
STAI	0.10	0.17	2.35*	
PISQ-12	−0.12	−0.13	−1.88	

*p < 0.05; **p < 0.01.

PISQ-12, Pelvic Organ Prolapse/Urinary Incontinence Sexual Questionnaire–12; PVD, provoked vestibulodynia; SF-12, health-related quality of life questionnaire; STAI, State-Trait Anxiety Inventory.

## Discussion

This study aimed to identify the demographic and clinical characteristics of women with PVD in Israel. Among the nearly 200 women who participated in this study, over a third reported symptoms of depression or anxiety, with most not receiving treatment. Additionally, many participants had other comorbidities, such as IBS, migraines, and fibromyalgia, which are often linked to depression and anxiety. Furthermore, one in four women reported experiencing sexual assault or domestic violence.

Consistent with previous research, our findings highlight the need for integrated care approaches that address both the physical and psychological aspects of PVD. The high prevalence of comorbid conditions such as IBS, migraines, and fibromyalgia among our participants suggests that PVD is often part of a broader spectrum of chronic pain syndromes. This underscores the importance of a multidisciplinary approach to treatment, incorporating both medical and psychological support to effectively manage the complex interplay of symptoms experienced by these patients.

According to the World Health Organization, the lifetime prevalence of intimate partner violence among women ranges from 13% to 61%.^16^ In Israel, Shwartz et al.^17^ reported that 36% of postpartum women experienced intimate partner violence. This high rate of intimate partner violence in our sample is consistent with these findings and does not distinguish our participants from other young women. The high prevalence of sexual assault and domestic violence among our participants underscores the critical need for trauma-informed care in the management of PVD. Addressing these traumatic experiences is essential for providing comprehensive and effective treatment for women suffering from PVD.

While almost two-thirds of the women with PVD reported primary vestibulodynia, the diagnosis process was notably prolonged, taking an average of 2.5 years and up to 6 years in some cases. This delay in diagnosis is consistent with other studies that highlight the challenges in recognizing and diagnosing PVD due to its multifactorial etiology and the lack of awareness among health care providers.^3^ The prolonged time to diagnosis emphasizes the need for better education and training for health care professionals to recognize and manage PVD more effectively.

All women included in our study sought treatment, with most trying multiple modalities. These findings highlight the urgent need for appropriate medical training in diagnosing vestibulodynia and improving the accessibility of information about vulvar pain syndromes. Despite the efforts to find relief, the majority of available treatments were largely ineffective. Physical therapy emerged as the most commonly used treatment. According to our data, 19% of women reported no improvement with physical therapy, while over two-thirds experienced some level of improvement. These results underscore the necessity for developing new and more effective treatment options for PVD.

Among the predictors of PVD severity, the age at which one begins sexual activity was found to be uniquely significant. The significant association between early sexual activity and the severity of PVD symptoms suggests that early intervention and education may be crucial in mitigating the long-term impact of this condition. It is possible that women who begin sexual activity at an older age are more physically and mentally mature, which may not always be the case for younger women. However, evaluating women’s physical and mental maturity was beyond the scope of this study and could be an interesting direction for future research.

Multidisciplinary treatment programs for PVD have shown promise in improving outcomes for affected women. These programs typically combine medical, psychological, and physical therapy interventions to address the various aspects of the condition.^18^ Studies have demonstrated significant improvements in sexual distress and pain following multidisciplinary treatment, with higher baseline levels of sexual desire and arousal predicting greater improvements.^18^ However, the literature on the effectiveness of these programs is still limited, and more research is needed to evaluate their long-term benefits and identify the most effective components.

### Study limitations

Our study has several limitations. First, the online data collection method excluded women without internet access, potentially limiting the generalizability of our findings. Additionally, our sample consisted only of women who were already diagnosed with PVD, excluding those who may be undiagnosed. Given the high rate of undiagnosed PVD, future research should explore physicians’ knowledge and willingness to diagnose this condition.

The study focused solely on women currently experiencing PVD, not including those who had recovered or had not yet been diagnosed. Randomized controlled trials are necessary to determine the effectiveness of various interventions under different conditions, and new therapies need to be developed and tested.

An additional limitation of this study is that we did not distinguish between primary and secondary PVD in the analyses. Future studies with larger sample sizes should examine potential differences between these groups.

Our study had a relatively small sample size, which may have limited our ability to identify other predictors with small effect sizes. Furthermore, the self-selected nature of our participants may not be representative of the broader population. Women who chose to participate might differ from those who declined in terms of PVD severity and causal factors. Therefore, randomly recruited samples are required to generalize the findings to the wider population.

Last, while we used validated screening instruments with good psychometric properties to assess depression and anxiety, these tools rely on self-report measures. Clinical evaluations are necessary for accurate diagnoses.

## Conclusions

PVD is a prevalent and debilitating condition that significantly impacts the quality of life for many women. This study highlights the substantial delays in diagnosis and the limited effectiveness of conventional treatments, underscoring the need for improved diagnostic protocols and more effective therapeutic options.

The findings reveal that PVD is often accompanied by high rates of comorbid conditions such as depression, anxiety, and other chronic pain syndromes, which complicate its management. The significant association between early sexual activity, endometriosis, fibromyalgia, and the severity of PVD symptoms suggests that these factors should be carefully considered in both diagnosis and treatment planning.

Physical therapy emerged as the most effective treatment modality among the options evaluated, though its success was not universal. This indicates a need for the development and validation of new treatment approaches that can provide more consistent and substantial relief for PVD sufferers.

In conclusion, addressing the multifaceted nature of PVD requires a comprehensive approach that includes timely diagnosis, effective management of comorbid conditions, and the development of new, evidence-based treatments. Enhancing awareness and education among health care providers and patients is essential to improving outcomes for women affected by this challenging condition.

Future research should focus on larger, more diverse populations to better understand the predictors and mechanisms underlying PVD. Additionally, there is a critical need for randomized controlled trials to identify the most effective interventions and to explore the potential benefits of integrated, multidisciplinary treatment strategies.

## Abbreviations Used

BMI: Body Mass Index
IBS: Irritable Bowel Syndrome
PVD: Provoked Vestibulodynia
SNRI: Serotonin Norepinephrine Reuptake Inhibitor
SSRI: Selective Serotonin Reuptake Inhibitor
